# The kinase occupancy of T cell coreceptors reconsidered

**DOI:** 10.1073/pnas.2213538119

**Published:** 2022-12-01

**Authors:** Alexander M. Mørch, Falk Schneider, Edward Jenkins, Ana Mafalda Santos, Scott E. Fraser, Simon J. Davis, Michael L. Dustin

**Affiliations:** ^a^Kennedy Institute of Rheumatology, University of Oxford, Oxford OX3 7FY, United Kingdom; ^b^Medical Research Council Human Immunology Unit, and Medical Research Council Weatherall Institute of Molecular Medicine, John Radcliffe Hospital, University of Oxford, Oxford OX3 9DS, United Kingdom; ^c^Translational Imaging Center, University of Southern California, Los Angeles, CA 90089

**Keywords:** coreceptor, kinases, fluorescence, signaling, diffusion

## Abstract

The sensitivity of the αβ T cell receptor (TCR) is enhanced by the coreceptors CD4 and CD8αβ, which are expressed primarily by cells of the helper and cytotoxic T cell lineages, respectively. The coreceptors bind to major histocompatibility complex (MHC) molecules and associate intracellularly with the Src-family kinase Lck, which catalyzes TCR phosphorylation during receptor triggering. Although coreceptor/kinase occupancy was initially believed to be high, a recent study suggested that most coreceptors exist in an Lck-free state, and that this low occupancy helps to effect TCR antigen discrimination. Here, using the same method, we found instead that the CD4/Lck interaction was stoichiometric (~100%) and that the CD8αβ/Lck interaction was substantial (~60%). We confirmed our findings in live cells using fluorescence cross-correlation spectroscopy (FCCS) to measure coreceptor/Lck codiffusion in situ. After introducing structurally guided mutations into the intracellular domain of CD4, we used FCCS to also show that stoichiometric coupling to Lck required an amphipathic α-helix present in CD4 but not CD8α. In double-positive cells expressing equal numbers of both coreceptors, but limiting amounts of kinase, CD4 outcompeted CD8αβ for Lck. In T cells, TCR signaling induced CD4/Lck oligomerization but did not affect the high levels of CD4/Lck occupancy. These findings help settle the question of kinase occupancy and suggest that the binding advantages that CD4 has over CD8 could be important when Lck levels are limiting.

## Significance

CD4 and CD8αβ are archetypal coreceptor proteins that potently enhance T cell antigen sensitivity but how they function is still debated. A fundamental question that remains incompletely resolved is: what fractions of the coreceptors bind the signal-initiating kinase, Lck? Using in vitro assays and noninvasive fluorescence cross-correlation spectroscopy, we show that most coreceptors are occupied by Lck at the surface of live cells. The structural basis for important differences in the kinase occupancy of CD4 and CD8αβ is also identified. These results provide important context for refining current models of both TCR antigen recognition and cell fate decisions made during thymopoiesis.

Conventional αβ T cells are divided into two major functional subsets depending on which of the two coreceptors they express, CD4 or CD8, that recognize class II and class I major histocompatibility complex (MHC) proteins, respectively. CD4^+^ T cells provide “help” to antibody-producing B cells by secreting cytokines, whereas CD8^+^ T cells are directly cytotoxic (1). The coreceptors increase the sensitivity of T cell receptor (TCR) signaling through recruitment of the Src-family kinase Lck, which is especially important for the recognition of low-affinity antigens (2, 3). Ablation of the Zn^2+^ clasp, which couples the coreceptor to Lck, results in severely diminished responsiveness to in vitro stimulation (4, 5). An explanation for this effect is that TCR triggering is a two-step process requiring initial incipient phosphorylation of the TCR by free Lck, which is very quickly followed by the recruitment of a coreceptor/Lck complex. This second step is thought to occur through bidentate interactions of the coreceptor/Lck complex with the TCR (via Lck) and its pMHC ligand, which further enhances receptor phosphorylation (6–8). However, it is also possible that, rather than increasing the sensitivity of signaling, the coreceptors effect antigen discrimination. According to this view, prompted by measurements by Stepanek et al. suggesting that limiting amounts of Lck occupy a small fraction of coreceptors, binding of cognate pMHC to the TCR is followed by processive coreceptor “scanning” until Lck is corecruited, favoring the phosphorylation of long-lived TCR complexes (9).

An important differentiator between these proposals is the extent to which coreceptors are bound to Lck, but a consensus is yet to emerge on this matter (10). Early coimmunoprecipitation (co-IP) experiments suggested that CD4/Lck occupancy is high (~90%) in mature T cells (11) but lower in thymocytes (25 to 50%) (12). More recent flow cytometric co-IP (FC-IP) assays from Stepanek and colleagues suggest an even lower thymocyte CD4/Lck occupancy (6%), leading to the proposal of “coreceptor scanning”. However, these FC-IP assays also contradicted the early reports, suggesting that only 37% of CD4 is Lck-occupied in mature T cells (13). Knowing the kinase occupancy level of CD4 and CD8αβ is critical for our understanding of TCR triggering during thymic development and mature T cell activation, prompting us to revisit this question with both FC-IP and in situ measurements.

Here we report, using FC-IP, that CD4 and CD8αβ have a high kinase occupancy (100% and 55%, respectively) in primary thymocytes. To confirm and extend these findings, we used scanning fluorescence cross-correlation spectroscopy (sFCCS) (14, 15) to measure coreceptor/Lck association in situ in live HEK293T and T cells. We found that the Lck occupancy of CD4 is high (100%) at the cell surface, and that this efficient coupling depends both on the conserved Zn^2+^ clasp motif and the amphipathic helix in the intracellular domain (ICD) of CD4 (16). CD8αβ lacks an amphipathic helix and, accordingly, exhibits a measurably lower occupancy (60%). Consistent with this difference, when the coreceptors were coexpressed in HEK293T cells with limiting amounts of kinase, CD4 outcompeted CD8αβ for Lck. Lastly, we present evidence that CD4/Lck occupancy is unaffected by early TCR signaling, indicating that coreceptor/Lck complexes are readily available prior to and during T cell activation.

## Results

### FC-IPs Indicate High Coreceptor/Lck Occupancy in Thymocytes and Jurkat T Cells.

**Fig. 1.**
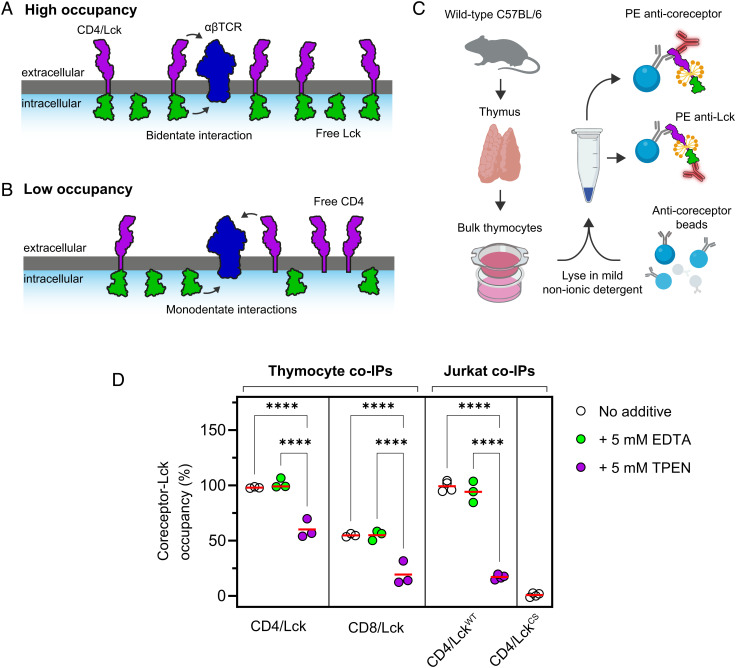
High coreceptor/Lck occupancy in both thymocytes and Jurkat T cells is only partially disrupted by chelation. *A*. and *B*. Depiction of high or low coreceptor occupancy at the T cell surface. At high occupancy (*A*), CD4/Lck complexes can form bidentate interactions with TCR/pMHC via Lck binding to phosphorylated ITAMs and CD4 binding to MHC-II (not depicted). At low occupancy (*B*), CD4 and Lck bind independently to TCR/pMHC complexes. Protein models based on crystal structures of CD4, Src, and αβTCR (PDB IDs: 1WIQ, 3EL8 and 6JXR, respectively). *C*. Schematic of the FC-IP assay used in this study, created with BioRender. *D*. FC-IP measurements of coreceptor/Lck occupancy in bulk thymocytes and Jurkats expressing murine (m)CD4 and mLck (*SI Appendix*, Fig. S1*C*) indicate that CD4 and CD8αβ form high-occupancy complexes with Lck independently of EDTA. TPEN partially dissociates the interaction, but there is a residual TPEN-resistant component in both thymocytes and Jurkat T cells. Each circle indicates a flow cytometry measurement from either one mouse or a replicate measurement of the indicated Jurkat T cell line. 10^7^ cells were lysed for each experiment. Clasp-deficient Lck contains the C20S and C23S mutations (Lck^CS^). Significance testing was performed with a one-way ANOVA followed by Tukey’s correction for multiple comparisons, and only significant comparisons are shown. ****P < 0.0001.

CD4/Lck occupancy measurements (Fig. 1 *A* and *B*) have traditionally been performed using co-IP assays in buffers that contain chelating agents, such as ethylenediaminetetraacetic acid (EDTA), to inhibit metalloprotease activity (9, 11, 17). However, EDTA has also been shown to disrupt the Zn^2+^-dependent association of CD4 and Lck (18), which might explain some of the discrepancies between reports. To determine whether the presence of Zn^2+^ chelators could affect the measured occupancy, we performed the FC-IP assay developed and used by Stepanek et al*.* (Fig. 1*C* and *SI Appendix*, Fig. S1*A*) (9). Thymocyte lysates from wild-type C57Bl/6 mice were incubated with beads conjugated to anti-mouse (m)CD4 antibodies before staining the beads for either mCD4, mouse (m)Lck or rat CD48 as a negative control (Fig. 1*D*). Residual bead fluorescence is measured with flow cytometry, and the background-subtracted fluorescence ratio between Lck and CD4 offers a measure of the coreceptor/Lck coupling ratio. Since the majority of thymocytes are CD4^+^ CD8αβ^+^ double-positive (DP) (19), we expected this assay to produce a low CD4/Lck occupancy (6 to 17%) as reported in experiments with sorted DP thymocytes (9, 13). Instead, however, we found that the interaction was effectively saturated (i.e.*,* all CD4 molecules were bound to Lck) (Fig. 1*D* and *SI Appendix*, Fig. S1*B*). The addition of EDTA to the lysates had no effect, implying that the detergent-solubilized complexes are resistant to Zn^2+^ chelation under these conditions. Pretreatment of cells with millimolar amounts of a membrane-permeable Zn^2+^ chelator, *N*,*N*,*N′*,*N′*-tetrakis(2-pyridinylmethyl)-1,2-ethanediamine (TPEN), which was also expected to disrupt the CD4/Lck interaction in vitro (20, 21) reduced the occupancy by ~40% (Fig. 1*D*). CD8αβ was substantially occupied by Lck (~55%), and this was reduced in the presence of TPEN treatment of live cells, but not EDTA treatment of lysates. This confirmed that sequestering Zn^2+^ in situ could disrupt the coreceptor/Lck interaction, but the residual association of precipitated proteins suggested either incomplete Zn^2+^ chelation by TPEN or the Zn^2+^-independent association of coreceptors and Lck captured in detergent micelles during lysis.

To test if the partial dissociations were due to nonspecific interactions, we used lentiviral gene delivery to transduce mCD4 and mLck into Jurkat T cells in which expression of endogenous CD4/Lck had been abolished using CRISPR/Cas9 (*SI Appendix*, Fig. S1*C*). We observed a high CD4/Lck occupancy for the wild-type interaction irrespective of EDTA treatment but TPEN substantially dissociated the complex (Fig. 1*D*). When the Zn^2+^ clasp of Lck was mutated (Lck^C20S, C23S^ = Lck^CS^), no Lck was precipitated, consistent with previous data showing that the clasp is essential for binding (22). These results lead us to conclude that TPEN was only partially chelating Zn^2+^ in the FC-IP assay, but indicated that the high occupancies observed in thymocytes were not an artifact of the in vitro solubilization method. However, the recent reports of low kinase occupancy (9) cannot be explained by the presence of EDTA in the solubilization buffer. To confirm our observations, and to resolve this discrepancy, we sought noninvasive approaches to measure the stoichiometry of coreceptor/Lck complexes at the surface of live cells.

### Analysis of CD4 and Lck Occupancy in Live Cells.

**Fig. 2.**
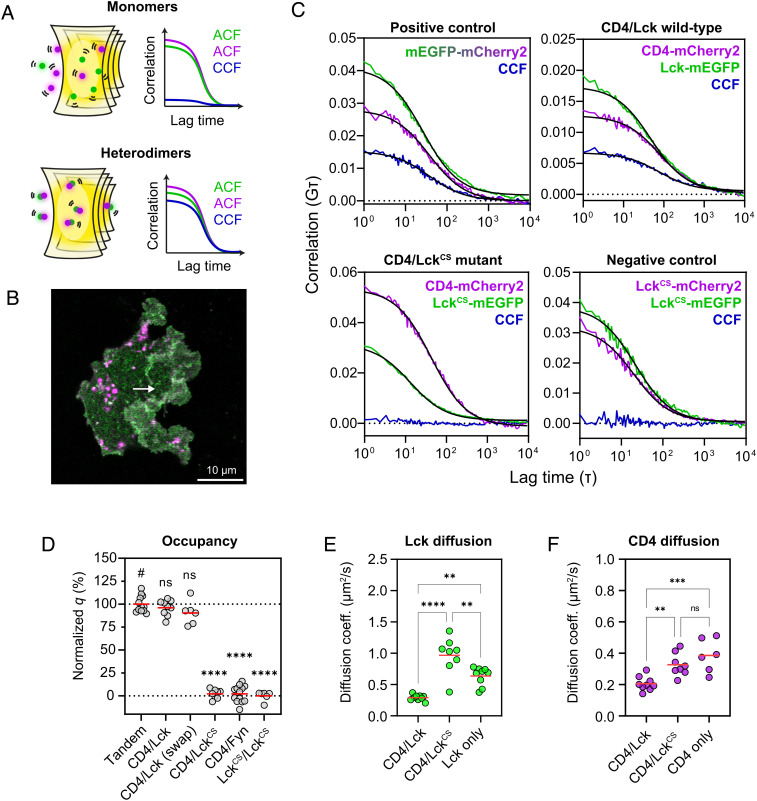
Scanning-FCCS measurements reveal a high CD4/Lck occupancy. *A*. Schematic of scanning-FCCS (sFCCS) volumes and correlation curves from monomeric (*Top*) and dimeric (*Bottom*) species. The ACFs (magenta and green) are identical for monomers and dimers and can be fitted to extract the diffusion terms. The CCF (blue) are different between monomers and dimers with a low amplitude (*Top*) indicating no codiffusion and a high amplitude (*Bottom*) indicating complete codiffusion. *B*. Representative confocal image of a HEK293T cell cotransfected with CD4-mCherry2 and Lck-mEGFP acquired in photon-counting mode. sFCCS measurements are acquired in a similar fashion with shorter pixel dwell times to sample fluorescence fluctuations. The length and position of a typical sFCCS line-scan is depicted by the white arrow. *C*. Representative line-averaged ACFs (magenta/green) and CCFs (blue) from one cell for each condition shown. Cross-correlation amplitudes are high for the positive control and wild-type CD4/Lck (*Top*), indicating that all CD4 molecules are bound to Lck. Also shown are negative control conditions with zero cross-correlation amplitudes (*Bottom*). Fits are shown in solid black lines. *D*. Cross-correlation quotients (*q*) for each condition normalized to the tandem control (100% co-diffusion) and Lck^CS^/Lck^CS^ (0% co-diffusion) with significant differences shown compared with the tandem (#), showing that the Lck clasp cysteines are essential for the high CD4/Lck occupancy. "CD4/Lck (swap)" indicates CD4-mEGFP coexpressed with Lck-mCherry2. *E*. and *F*. Diffusion coefficients for Lck-mEGFP (green) and CD4-mCherry2 (magenta) derived from the fitted ACFs showing a significant increase in the diffusion speed of both CD4 and Lck when the Zn^2+^ clasp is disrupted. In *D*, *E*, and *F* each circle represents a spatially averaged line-scan measurement from one cell. Cells were pooled from three independent replicates for 6 to 13 cells per condition. Significance testing was performed with a one-way ANOVA followed by Dunnett’s correction in *D* and Tukey’s correction in *E* and *F* for multiple comparisons. ****P < 0.0001, ***P < 0.001, **P < 0.01.

Resonance energy transfer experiments indicate that CD4 is invariably bound to Lck in transfected cells (23) and, similarly, fluorescence microscopy shows Lck is efficiently cocapped with either CD8αα or CD8αβ (24). However, there have been no absolute measurements of coreceptor/Lck occupancy at the surface of live cells. We therefore employed fluorescence cross-correlation spectroscopy (FCCS), a two-color fluctuation spectroscopy method developed to quantify molecular dynamics in situ (25, 26) (Fig. 2*A*), to measure the codiffusion of CD4 and Lck as proof-of-concept. The autocorrelation functions (ACFs) generated by FCCS provide information on diffusion and density, while cross-correlation functions (CCFs) measure heterotypic interactions between spectrally distinct labels (15). In order to perform FCCS of membrane proteins, which diffuse slowly and are prone to photobleaching, we acquired fluorescence signals in scanning-mode (sFCCS) which scans multiple contiguous pixels sequentially to improve signal-to-noise and reduce photobleaching effects (*SI Appendix*, Fig. S2*A*) (27). To this end, full-length human CD4 and Lck constructs were expressed with appropriate fluorescent protein (FP) tags (28) (mCherry2 and mEGFP, respectively) in HEK293T cells grown on glass coverslips (Fig. 2*B*). Intensity fluctuations were acquired using a confocal microscope in photon-counting mode and correlation analyses were performed using FoCuS_scan software (29) (Fig. 2*C*). Using the calibrated size of the observation volume, we also determined fluorophore density (*SI Appendix*, *SI Materials and Methods*) and confirmed that all constructs were expressed at physiological levels similar to the normal densities of CD4 and Lck (*SI Appendix*, Fig. S2*B*). To control for underestimation of interactions that might result from nonfluorescent FPs, we normalized the experiments to a positive control consisting of the transmembrane domain of CD4 linked covalently to both mEGFP and mCherry2 (“Tandem”, Fig. 2*C*). Codiffusion of mEGFP and mCherry2, i.e.*,* the cross-correlation quotient (*q*, *SI Appendix*, *SI Materials and Methods*), was then determined as the ratio between the CCF amplitude and the minimum ACF amplitude (26).

Wild-type CD4 and Lck produced a very high *q* value (96% ± 8%), irrespective of which FP was conjugated to either protein (Fig. 2*D*), indicating that essentially all CD4 was codiffusing with Lck. To rule out any effects of nonspecific association, we generated three different negative controls: CD4/Lck^CS^, CD4/Fyn, and Lck^CS^/Lck^CS^. In Lck^CS^, the clasp cysteines essential for binding CD4 are mutated to serine to abolish the interaction (22). Fyn is a Src-family kinase that is structurally similar to Lck (30) but cannot interact with coreceptors (31). In the last condition, we expected the coexpression of Lck^CS^-mEGFP and Lck^CS^-mCherry2 to control for any lipid-mediated targeting as this form of Lck cannot homodimerize (32), but the lipid attachment sites remain intact. No cross-correlation was detected in any of the negative controls, confirming that the high CD4/Lck occupancy was specific only to an intact Zn^2+^ clasp (Fig. 2*D*).

While CD4 is a transmembrane protein, Lck is only anchored to the inner leaflet through lipid-modified residues in the SH4 domain (33). Given the high CD4/Lck occupancy, this suggested that the diffusion of coreceptor-bound Lck would be significantly slower than free Lck according to the Saffman–Delbrück model of diffusion in biological membranes (34–36). To test this, we used sFCCS to determine the diffusion coefficients of CD4/Lck, CD4/Lck^CS^, and CD4/Lck expressed alone (refer to *SI Appendix*, *SI Materials and Methods* for transit time calculations). Wild-type CD4 and Lck exhibited similar diffusion coefficients (Lck = 0.29 ± 0.05 µm^2^/s, CD4 = 0.21 ± 0.05 µm^2^/s), which increased significantly upon mutation of the Lck clasp (Lck^CS^ = 0.97 ± 0.29 µm^2^/s, CD4 = 0.33 ± 0.07 µm^2^/s), indicating that disrupting the interaction increased the diffusion rates of both CD4 and Lck (Fig. 2 *E* and *F*). In the case of CD4, this diffusion was comparable to when the protein was expressed alone (0.39 ± 0.11 µm^2^/s). When Lck was expressed alone, its diffusion speed was substantially reduced compared with Lck^CS^ (0.64 ± 0.15 µm^2^/s), and we speculate that this might be due to weak Lck homodimerization through the clasp (32). Our diffusion coefficients differ somewhat from those reported previously using other biophysical methods (37, 38), a difference that may be attributed to the challenges of performing fluctuation spectroscopy in heterogeneous and undulating plasma membranes (39–41). However, the relative changes in diffusion allowed us to conclude that the bulk of CD4 molecules codiffuse with Lck in the steady state.

### The CD4 Amphipathic Helix Is Necessary for Efficient Lck Association.

**Fig. 3.**
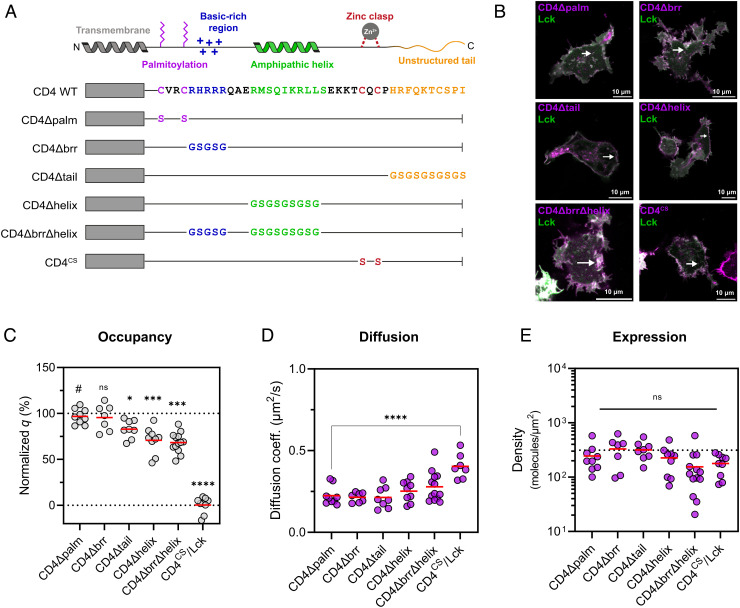
Contributions of CD4 sequence motifs to efficient Lck association. *A*. Schematic of the panel of CD4 mutants used to test the binding contributions of the intracellular sequence elements indicated at the top. *B*. Confocal images of HEK293T cells coexpressing the indicated mCherry2-tagged CD4 constructs with wild-type Lck-mEGFP acquired in photon-counting mode with the white arrows indicating the approximate position of the line-scan. *C*. The CD4 amphipathic α-helix makes an important contribution to the stoichiometric codiffusion of CD4/Lck complexes. Cross-correlation quotients (*q*) were normalized to the controls in Fig. 2; statistical comparisons were made to the CD4Δpalm condition (#). *D*. CD4 diffusion coefficients are similar between constructs with an increase seen only on the loss of Lck-binding; statistical comparisons as in *C*. *E*. All CD4 constructs express at similar molecular densities. The dotted line indicates the average expression density of wild-type CD4 from *SI Appendix*, Fig. S2*B*. In *C*, *D*, and *E* each circle represents a spatially averaged line-scan measurement from one cell, with measurements pooled from three independent replicates for 7 to 13 cells per condition. Significance testing was performed with a one-way ANOVA followed, in *C* and *D*, by Dunnett’s correction for multiple comparisons. ****P < 0.0001, ***P < 0.001, **P < 0.01, *P < 0.05.

There is evidence from early mutagenesis studies that intracellular sequence elements in CD4 other than the Zn^2+^ clasp might contribute to the interaction with Lck (22, 42). When overlaid with the CD4/Lck NMR structure (16), these elements appear to fall into three regions: the membrane-proximal basic-rich region (BRR), the amphipathic α-helix, and the unstructured C-terminal “tail.” To analyze the contributions of these features of CD4, we generated a panel of full-length CD4 molecules each bearing mutations in either the BRR, the helix, or the tail (Fig. 3*A*). To avoid making large truncations that might alter the spacing between the plasma membrane and the Zn^2+^ clasp, we replaced each sequence of interest with a flexible (Gly-Ser)_n_ linker of equivalent length. Each mCherry2-tagged CD4 construct was then coexpressed with wild-type Lck-mEGFP in HEK293T cells for sFCCS measurements (Fig. 3*B*). We found that, while the BRR did not contribute to Lck association, mutation of the amphipathic helix reduced CD4/Lck occupancy by 27% (Fig. 3*C*). When both were mutated simultaneously, occupancy was reduced by 28% indicating a significant role for the amphipathic helix in efficient CD4/Lck complex formation. As controls, we mutated the CD4 palmitoylation sites in CD4 (CD4Δpalm) which had no effect on CD4/Lck occupancy (43) or the clasp cysteines (CD4^CS^), which abolished the interaction (Fig. 3*C*). This demonstrated that an intact Zn^2+^ clasp is necessary but insufficient for the formation of high-occupancy CD4/Lck complexes. Additionally, mutation of the unstructured tail produced a small (~14%) decrease in occupancy (Fig. 3*C*). This disordered C terminus is absent from the CD4/Lck NMR structure (16) but may be involved in stabilizing the clasp by interacting with proximal elements of Lck. To confirm that the effects on occupancy were not due to inadvertent effects on mobility or expression, we then used FCCS to determine diffusion coefficients and fluorophore density. None of the CD4 constructs diffused significantly faster than the positive control (CD4Δpalm), except when Lck-binding was completely abolished (CD4^CS^) (Fig. 3*D*) as expected from similar measurements in Fig. 2*F*. In addition, each construct expressed at levels similar to wild type (dotted line) demonstrating no changes in folding efficiency (Fig. 3*E*). Overall, our mutation experiments show that the wild-type, stoichiometric interaction of CD4 with Lck relies on both the Zn^2+^ clasp and the amphipathic helix of CD4.

### CD4 Outcompetes CD8αβ for Lck Binding in Dual Coreceptor-Expressing Cells.

**Fig. 4.**
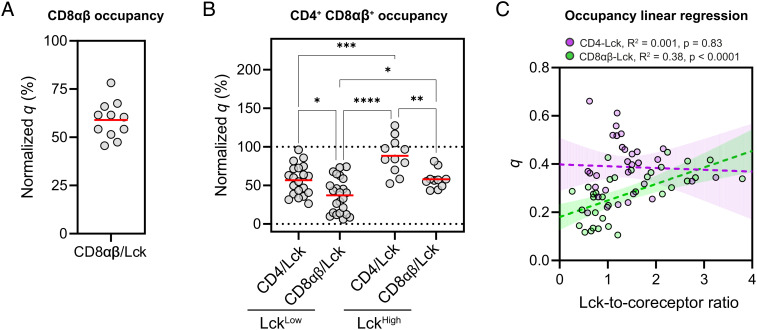
CD4 outcompetes CD8αβ for Lck at limiting kinase levels. *A*. sFCCS measurements of CD8αβ/Lck were performed by transfecting CD8α, CD8β/mCherry2, and Lck-mEGFP into HEK293T cells and normalizing *q* values to the controls in Fig. 2, yielding an occupancy of 59% ± 10%. *B*. Dual coreceptor-expressing HEK293Ts were generated by transfecting either CD4-mCherry2 and unlabeled CD8α and CD8β, or CD8β/mCherry2 and unlabeled CD4 and CD8α. Either 25 ng or 50 ng Lck-mEGFP plasmid DNA was cotransfected to generate the Lck^Low^ or Lck^High^ conditions, respectively. The *q* values were determined as in *A*. In dual coreceptor-expressing Lck^Low^ cells, occupancy is lower than in cells expressing just one coreceptor with a larger variance, suggesting that CD4 and CD8 compete for limiting Lck in individual cells. When Lck is in excess (Lck^High^), occupancy is restored to that of SP cells indicating saturation of both coreceptors. *C*. Linear regression was used to test for a link between the amount of excess Lck (Lck/coreceptor ratio) and kinase occupancy for both CD4/Lck (magenta) and CD8αβ/Lck (green). Only the latter showed a positive, significant relationship indicating that CD4 outcompetes CD8αβ for Lck when it is limiting. F-tests were used to determine whether the slopes differed significantly from zero. Dotted lines indicate regression slopes, and shaded areas indicate 95% CI. In each panel, a circle represents a spatially averaged line-scan measurement from one cell, with measurements pooled from three independent replicates for 10 to 23 cells per condition. Significance testing was performed with a one-way ANOVA followed by Tukey’s correction for multiple comparisons. ****P < 0.0001, ***P < 0.001, *P < 0.05.

CD8/Lck interactions are weaker than CD4/Lck interactions in co-IP assays (Fig. 1*D*), and there is a twofold difference in affinities when assayed as isolated polypeptides in solution (16), perhaps reflecting the lack of known secondary structure in the cytoplasmic domain of CD8α. We cotransfected CD8α, CD8β-mCherry2, and Lck-mEGFP into HEK293T cells and found that the CD8αβ/Lck occupancy was 59% ± 10%, similar to that of helix-deficient CD4 (Fig. 4*A*). The high occupancy of both coreceptors also suggested that they might compete for Lck when coexpressed in the same cell. To test this possibility, we cotransfected CD4-mCherry2 with nonfluorescent CD8α/CD8β or CD8β-mCherry2 with nonfluorescent CD8α/CD4, alongside equivalent amounts of Lck-mEGFP, and measured the coreceptor/Lck *q* under each condition (Fig. 4*B*). The average occupancies of both CD4/Lck (57% ± 20%) and CD8αβ/Lck (37% ± 20%) in dual coreceptor-expressing cells were substantially reduced compared with when a single coreceptor was expressed (cf*.* 96% for CD4/Lck, 59% for CD8αβ/Lck Figs. 2*D* and 4*A*), implying that all available kinase was partitioned between coreceptors when Lck was limiting (Lck^Low^). We hypothesized, therefore, that increasing Lck levels in excess of CD4 and CD8 (Lck^High^) would reverse this trend, restoring the coreceptor/Lck equilibrium to that of cells with just the one coreceptor. These measurements proved to be technically challenging because increased mEGFP expression led in most cases to cell brightness beyond the range for single-molecule fluctuation measurements. Nonetheless, in the narrow range of cells yielding suitable fluctuation curves, increased Lck-mEGFP expression levels (*SI Appendix*, Fig. S3) yielded average occupancy values comparable to single coreceptor-expressing HEKs for both CD4 (88% ± 23%) and CD8αβ (60% ± 12%) (Fig. 4*B*). This suggested that Lck expression levels could regulate coreceptor occupancy, with CD4 outcompeting CD8αβ when Lck is limiting. To test this statistically, we pooled Lck^Low^ and Lck^High^ cells and used linear regression to analyze the relationship between the amount of excess Lck (i.e.*,* the Lck/coreceptor density ratio) and kinase occupancy (Fig. 4*C*). Although there was no statistical link between the CD4/Lck *q* and the Lck/coreceptor ratio (R^2^ = 0.001, *P* = 0.83), CD8/Lck *q* increased significantly with excess Lck (R^2^ = 0.38, *P* < 0.0001). At low Lck concentrations, CD4 outcompetes CD8αβ, and it is only when the Lck levels begin exceeding both coreceptors (Lck/coreceptor ratio ≥ 2) that CD8αβ/Lck approaches the occupancy observed in cells expressing CD8αβ alone. Together, our results indicate that the coreceptor/Lck equilibrium in dual coreceptor-expressing cells is sensitive to Lck expression.

### CD4/Lck Occupancy Is Independent of TCR Signaling.

**Fig. 5.**
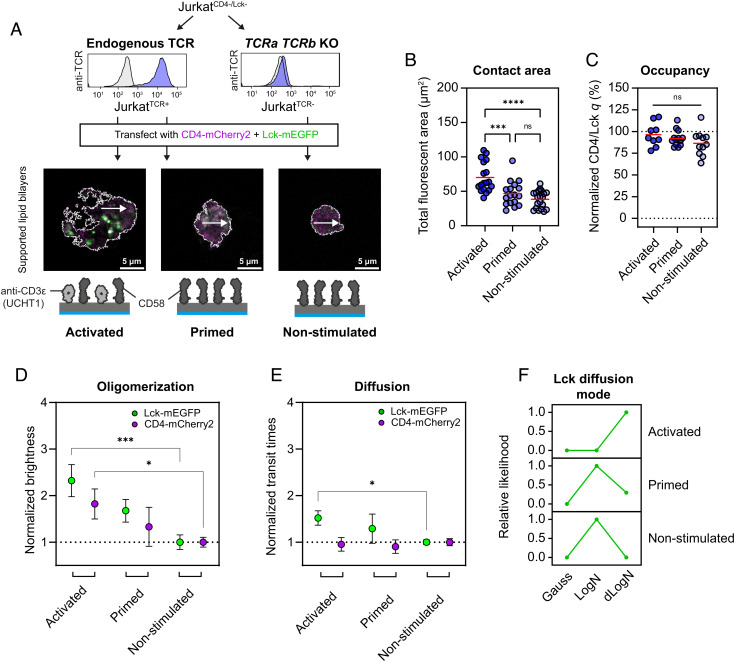
CD4/Lck occupancy is stable during early TCR signaling. *A*. TCR expression was ablated in Jurkat^CD4−/Lck−^ cells to produce Jurkat^TCR−^ cells, assayed with flow cytometry (*Top*) using anti-TCR antibodies conjugated to PE (blue). Fluorescence levels of Jurkat^TCR−^ cells match the isotype control (gray), confirming knockout. Confocal images (*Bottom*) of Jurkats cells cotransfected with CD4-mCherry2 (magenta) and Lck-mEGFP (green) and plated onto SLBs functionalized with recombinant His-tagged human proteins. CD58 (dark gray, 200 molecules/µm^2^) was used to anchor the Jurkat T cells to the surface and anti-CD3ε Fab (light gray, clone UCHT1, 30 molecules/µm^2^) was used for activation of the TCR. Three conditions were tested: Jurkat^TCR+^ cells on UCHT1 + CD58 SLBs (activated), Jurkat^TCR+^ cells on CD58 SLBs (primed), and Jurkat^TCR−^ cells on CD58 SLBs (nonstimulated). The white lines indicate the outline of the cell based on fluorescence thresholding. Protein models were based on the crystal structures of a Fab fragment and hCD58 (PDB IDs: 6TCR and 1CCZ, respectively). *B*. The average spreading areas of Jurkats cells in the activated, primed, and nonstimulated conditions, as measured using the total fluorescent area for each cell, show an increase with TCR stimulus. Each circle indicates one cell with measurements pooled from three independent replicates for 17 to 20 cells per condition. *C*. CD4/Lck cross-correlation quotients (*q*) normalized to the controls in Fig. 2, indicating that CD4/Lck occupancy does not change between conditions. Each circle indicates a spatially averaged line-scan measurement from one cell, with measurements pooled from three independent replicates for 9 to 11 cells per condition. *D*. and *E.* Population-level brightness (*D*) and transit time (*E*) values for the three signaling conditions, normalized to the nonstimulated setting, show an increase in oligomerization (brightness) and no major changes in diffusion speed across conditions. The median brightness/transit time was calculated for each replicate (i.e.*,* all line-scan values for all cells on one day of measurements), and the mean value of three replicates is indicated with the circle, with error bars indicating one SD. Significance testing in both *D* and *E* was performed with a one-way ANOVA followed by Sidak’s correction for multiple comparisons of the same protein (i.e.*,* only Lck or only CD4) between activation conditions. Nonsignificant comparisons are not shown. *F*. Statistical analysis of Lck-mEGFP transit time histograms shows that Lck diffuses freely in the primed and nonstimulated conditions, but adopts a hindered diffusion mode in the activated condition only. Using an MLE approach, hindrances in diffusion can be revealed by histogram fitting. The relative likelihood value for the model representing the data best is 1. Fluorescently labeled molecules diffusing freely follow a lognormal distribution (LogN) and molecules that undergo nanoscale trapping interactions result in a double-lognormal distribution (dLogN). Significance testing in *B* and *C* was performed with a one-way ANOVA followed by Tukey’s correction for multiple comparisons. ****P < 0.0001, ***P < 0.001, *P < 0.05.

Our experiments in HEK293T cells were designed to measure in situ coreceptor/Lck interactions in the absence of any T cell-specific factors, but an early hypothesis for coreceptor function was that the activities of CD4 and CD8αβ could be controlled by TCR signaling (44) potentially through regulating kinase occupancy (45). To track coreceptor/Lck interactions in the context of TCR triggering, we transfected CD4-mCherry2 and Lck-mEGFP into Jurkat^CD4-/Lck-^ T cells (*SI Appendix*, Fig. S1*C*) and incubated them at 37 °C on supported lipid bilayers (SLBs) functionalized with the small adhesion protein CD58. CD58 was necessary to induce Jurkat T cells to form suitably stable contact areas for sFCCS, as they would otherwise migrate or drift (46) and introduce movement artifacts into the fluctuation analysis. Then, to activate the cells through TCR engagement, anti-CD3ε (clone UCHT1) Fabs were tethered to the SLBs alongside CD58 (Fig. 5*A*, “activated”). To control for the basal level of activation that occurs through the CD58 condition alone (“primed”), owing to ligand-independent TCR triggering (47, 48), we generated TCR-deficient Jurkat^CD4−/Lck−^ cells (Jurkat^TCR−^) using CRISPR/Cas9 (Fig. 5*A*, “nonstimulated”). The diminished signaling capacity of Jurkat^TCR−^ cells was verified by Ca^2+^ flux measurements when placed onto SLBs decorated with UCHT1 Fabs and CD58 (*SI Appendix*, Fig. S4*A*). We noticed, additionally, that Jurkat^TCR−^ cells did not spread in the characteristic manner of activated T cells on SLBs (Fig. 5*A* and *SI Appendix*, Fig. S4*B*). This difference was confirmed by measuring the fluorescent area (i.e.*,* the area containing either CD4 or Lck) at the plane of contact with the SLB, and the average area was found to be significantly increased in the activated condition compared with either the primed or nonstimulated setting (Fig. 5*B*). CD4/Lck interactions were analyzed by performing sFCCS in the Jurkat T cells that had formed stable contacts within 10 min of incubation (Fig. 5*A*, white arrows). The cross-correlation analysis for these measurements produced high CD4/Lck *q* values, identical to the occupancies measured in HEK293T cells (Fig. 2*D*), indicating that signaling through the TCR had no effect (Fig. 5*C*). This implies that CD4 is invariably bound to Lck at the T cell surface, and that high occupancy is not an artifact of heterologous expression. These results are also consistent with our FC-IP data with Jurkat T cells expressing murine proteins (Fig. 1*C*), suggesting that CD4 and Lck are coupled stoichiometrically in both humans and mice.

T cell activation through the TCR leads to clustering of signaling proteins (49) and single-molecule imaging experiments have shown that both CD4 and Lck can form small, nanoscale clusters within minutes of triggering (50, 51), in part through confinement in CD2 nanodomains (52). To analyze population-level oligomerization effects (i.e.*,* integrated over all positions in all cells), we compared molecular brightness values (28) and found them significantly increased upon ligation of the TCR, indicating signaling-specific oligomerization of both CD4 and Lck (Fig. 5*D*). Smaller changes were seen in the bulk transit times of CD4 and Lck, with the only significant difference being faster Lck diffusion in the TCR-stimulated compared with the nonstimulated condition (Fig. 5*E*). Previous studies show, nonetheless, that the diffusion mode of Lck becomes “trapped” or hindered in activated T cells only (52–54). We therefore analyzed the scanning fluorescence correlation spectroscopy (sFCS) transit time histograms of Lck using maximum likelihood estimation (MLE) to determine if the histogram is best represented by a model for free diffusion or hindered diffusion, indicating nanoscale interactions (refer to *SI Appendix*, *SI Materials and Methods* for details) (55, 56). Using MLE analysis, we found that Lck exhibited free (lognormal) diffusion in the primed and nonstimulated states and trapped (double-lognormal) diffusion in the activated condition (Fig. 5*F*). Our results indicate that early TCR signaling has no effect on coreceptor occupancy, but does lead to changes in nanoscale organization in the form of transient hindrances producing trapped diffusion of Lck in the activated state only.

## Discussion

The ability of CD4 and CD8αβ to recruit kinase activity to the TCR, and their importance for potentiating T cell responsiveness, first became apparent more than 30 years ago (57, 58). It is surprising that the precise kinase occupancy of these proteins is still controversial despite being an important element of several TCR signaling models (9, 59–61). Our noninvasive sFCCS experiments showed that the coreceptors are coupled to Lck at high occupancy in situ, that the CD4/Lck stoichiometry relies on both the Zn^2+^ clasp and the amphipathic helix, and that kinase occupancy is unaffected by TCR signaling. The conclusion that the coreceptors are mostly occupied by Lck was supported by our FC-IP data and is consistent with earlier reports showing high levels of Lck in precipitates with anti-CD4 antibodies (17, 18, 20, 62–64). The finding that CD4/Lck and CD8/Lck occupancies are similarly high in both thymocytes and dual coreceptor-expressing HEKs with excess Lck also suggests that kinase occupancy in the thymus is unlikely to be as low as previously claimed (9, 12, 13). It is unclear why, using the same assay, i.e.*,* FC-IP, our results differ from those of prior studies (e.g.*,* 6%, 17% and 37% for CD4/Lck) (9, 13). One possibility is that there were differences in sample preparation. For example, the use of serum-free Ca^2+^-containing buffers might have caused Zn^2+^ efflux prior to lysis (65), which could have disrupted the coreceptor clasp. Another possibility is that excessive dilutions due to large (~1 ml) washing volumes (66) could have shifted the equilibrium of the complex toward dissociation. Kinetic analyses indicate that these interactions have relatively short half-lives (10, 16) which could yield different occupancies depending on factors such as antibody-bead coupling efficiency, duration of the pulldown step, number of washing steps, etc.

Although there is evidence that both CD4 and Lck can form homodimers when expressed alone (23, 32, 67), our sFCCS measurements show that the CD4/Lck heterodimer dominates when they are coexpressed, consistent with earlier resonance energy transfer measurements (23). In HEK293T cells, Lck expression was reduced when it could not bind CD4, implying that the trafficking of Lck is linked to CD4 association. Lck-deficient Jurkat T cells have been shown to up-regulate surface CD4 expression when transfected with an Lck transgene (68) and pulse–chase labeling experiments indicate that CD4/Lck complexes form rapidly after translation (69). This implies that CD4 and Lck form high-occupancy complexes prior to arriving at the cell surface. By introducing mutations into different regions of the CD4 ICD with reference to the NMR structure of the Zn^2+^ clasp, which was unavailable at the time of earlier mutagenesis experiments (16), we could also dissect contributions made by the ICD to association with Lck. This confirmed the important contribution of the CD4 intracellular helix and helped to explain why CD8αβ, which lacks the helix, binds Lck more weakly than CD4. Alignment of homologous mammal, bird, amphibian, and fish CD4 sequences indicates that residues in the amphipathic helix are highly conserved (10) – second only to the Zn^2+^ clasp cysteines – suggesting that there has been considerable evolutionary pressure to preserve this functionally important element of CD4.

How do our results relate to the roles of the coreceptors when CD4 and CD8αβ are coexpressed in thymocytes prior to lineage commitment (1)? It is unclear whether the coreceptors need only to sequester all the available Lck during this stage (60, 70) (i.e.*,* the “Lck availability” model), or if it is also important that coreceptors bind Lck at low capacity in order to effect differential signal amplification (9, 13) (i.e.*,* the “coreceptor scanning” model). The high kinase occupancy observed in our experiments with dual coreceptor-expressing cells argues against a low stoichiometry being an important contributor to antigen discrimination. A comprehensive analysis of TCR-pMHC binding studies shows that the increase in TCR sensitivity afforded by coreceptors comes at the cost of a reduction in discriminatory power, rather than increasing it (71). Differences between the levels of activity of Lck attached to the wholly occupied coreceptors could, in principle, contribute to proof-reading, but the catalytic activity among the differently phosphorylated (activity) states varies over a limited range (k_cat_/K_M_ = 0.23 × 10^−2^ µm^−2^ s^−1^ to 2.3 × 10^−2^ µm^−2^ s^−1^) (72).

To study the dynamics of CD4/Lck diffusion in the context of early TCR signaling, we used CD58-functionalized SLBs to create stable, nonsynaptic T cell contacts (47). Although CD4/Lck occupancy was independent of triggering, protein oligomerization could be detected through increased molecular brightness, in addition to the transition in Lck diffusion mode indicating that diffusion is hindered at the nanoscale. Stimulated emission depletion FCS measurements have previously shown that small, membrane-anchored enzymes like Lck exhibit trapped diffusion in “signaling nanoclusters” (73), and the formation of such nanoclusters likely precedes the nucleation of actin-dependent TCR microclusters in T cells (49, 52, 74). Only a small fraction of Lck molecules are fully active (i.e.*,* Y394-phosphorylated) in resting T cells (75), and super-resolution snapshots suggest this form of Lck does not colocalize with CD4 in clusters (76). This implies that the bulk CD4/Lck complexes detected in our experiments may contain Y394-unphosphorylated Lck, consistent with the recent finding that coreceptor-bound Lck is less active than free Lck (77).

Our observations help settle the question of how CD4 and CD8αβ associate with Lck in T cells, and offer additional support for the notion that the principal functions of coreceptors are to effect MHC restriction in the thymus and to amplify receptor signaling in the periphery (6, 44). Since both proteins serve such similar roles, why do they associate differentially with Lck? An important observation from our experiments with dual coreceptor-expressing HEKs is that, while both coreceptors are sensitive to Lck abundance, CD4-Lck occupancy is, on average, always higher than CD8αβ-Lck occupancy. Recent work by the Singer group has shown that DP thymocyte lineage fate is determined not by the nature of the coreceptor proteins, but rather by *cis* elements in the coreceptor loci that control their expression (78). We speculate that, during the early stages of signaling before changes in *CD4* and *CD8* transcription occur, the higher CD4/Lck occupancy versus that for CD8αβ compensates for the very low affinity of CD4/MHC II interactions (79) helping it match the signals generated by CD8αβ during thymopoiesis.

## Materials and Methods

The materials and methods used are summarized below with detailed information available in *SI Appendix*.

### co-IP.

FC-IP experiments were conducted as described in the published protocol (66). 10^7^ thymocytes from wild-type B6 mice were lysed at 4 °C for 30 min in lysis buffer before incubating with anti-mCD4 (clone RM4-4, BioLegend) or anti-mCD8β (clone 53-5.8, BioLegend). Zn^2+^ was chelated by pretreating cells with 5 mM TPEN or adding 5 mM EDTA to lysis buffers. Beads were washed and stained with fluorochrome-conjugated antibodies to Lck (3A5, SCBT), mCD4 (H129.19, BioLegend), mCD8α (53-6.7, BioLegend), or rat CD48 as a negative control (OX45, BioLegend). Residual bead fluorescence was measured by flow cytometry using an LSRFortessa^TM^ X-20 (BD Biosciences) and occupancy determined as the ratio of background-subtracted Lck fluorescence to coreceptor fluorescence adjusted for F/P ratios.

### Cell Culture and CRISPR-Cas9.

Jurkat E6.1 T cells and HEK293T cells (ATCC) were cultured in a complete R10 medium (Roswell Park Memorial Institute (RPMI) Medium-1640, 10% fetal bovine serum (FBS), 1% penicillin–streptomycin, 2% L-glutamine, 25 mM 4-(2-hydroxyethyl)-1-piperazineethanesulfonic acid (HEPES)) or a complete D10 medium (Dulbecco's Modified Eagle Medium (DMEM), 10% FBS, 1% penicillin/streptomycin), respectively, and maintained at 37 °C, 5% CO_2_ at appropriate densities. All cell culture reagents were purchased from Thermo Fisher Scientific unless otherwise indicated.

Gene expression was ablated using the LentiCRISPRv2 system with guides generated using Benchling (refer to *SI Appendix* for details on guide sequences and cloning). Lentiviruses were produced by transfecting 0.5 μg transfer plasmid plus 0.5 μg pMDG-VSVG and 0.5 μg pCMV-dR8.91 packaging plasmids into HEK293T cells using GeneJuice® (Novagen) for 72 h. The supernatant was 0.45 µm filtered and added directly to 10^6^ Jurkat cells for another 72 h before selection pressure was applied with 1 µg/ml puromycin (Sigma-Aldrich) for 7 d. Protein expression was examined by flow cytometry.

Stable expression (mCD4, mLck, mLck^CS^) was achieved with a lentiviral gene delivery system using a pHR-SIN transfer plasmid and the packaging plasmids described above. Transient transfections (hCD4, hLck, hCD4 mutants) were performed using a mixture of 100 ng plasmid DNA and 0.5 µl GeneJuice (Novagen), which was added to 5 × 10^4^ target cells the day before imaging, according to the manufacturer’s protocols. Refer to *SI Appendix* for a complete description of cloning strategies; briefly, murine sequences were obtained by PCR of cDNA gifted by members of the Davis lab, while human sequences were synthesized as cDNA fragments and purchased from Thermo Fisher Scientific). All inserts were subcloned into the pHR-SIN transfer plasmid and verified by sequencing (Source Bioscience).

### Flow Cytometry.

To assess the expression of surface or cytosolic molecules, 5 × 10^5^ cells were washed in flow cytometry buffer followed by staining with PE-conjugated anti-hCD4 (RPA-T4, BioLegend), anti-mCD4 (H129.19, BioLegend), or anti-αβTCR (IP26, BioLegend) at 10 µg/ml at 4 °C before fixation (4% formaldehyde in phosphate-buffered saline (PBS)). For intracellular staining, fixed cells were permeabilized with 0.5% saponin and stained with anti-Lck-PE (3A5, SCBT). Cells were washed twice before analysis at a flow cytometer.

### Imaging and sFCCS.

Imaging and sFCCS were performed on a Zeiss LSM 780 inverted confocal microscope (LSM780, Carl Zeiss) equipped with a 40× C-Apochromat 1.2 NA water-immersion FCS objective, hybrid GaAsP detectors, and a 488-nm Argon laser (for mEGFP excitation) and a 594-nm He–Ne Laser (for mCherry2 excitation). Point-FCS measurements were used to calibrate the system prior to each day of experiments using 20 nM Alexa Fluor 488 (Themo Fisher Scientific). sFCCS was performed in photon-counting mode and line scans positioned at the basal cell surface for fluorescence intensity measurements. Files were saved as .lsm5 files and correlated externally using FoCuS_scan (29). For details on the calculation of cross-correlation quotients and diffusion coefficients, refer to *SI Appendix*. Fluorescence images were also acquired in photon-counting mode and images processed with ImageJ (v1.53n, NIH) (80). Graphing and statistical analysis were performed with GraphPad Prism (v9.3).

### SLBs.

SLBs were prepared using liposome deposition according to previous protocols (81). Briefly, clean glass coverslips affixed with adhesive chambers (ibidi) were infused with DOPC vesicles supplemented with 12.5% DOGS-NTA (Avanti Polar Lipids) before washing with HEPES-buffered saline (HBS) containing 0.1% human serum albumin (HSA, Merck-Millipore). SLBs were blocked with 5% BSA and 100 µM NiSO_4_ before being functionalized with His-tagged proteins (anti-CD3ε UCHT1-Fab at 30 molecules/µm^2^ and CD58 at 200 molecules/µm^2^, both produced in-house). Unbound proteins and liposomes were removed with extensive HBS/HSA washing. Protein densities were calibrated using bead-supported bilayers loaded with FPs with reference to calibration beads (Bangs Laboratories).

### Calcium Release Assay.

For calcium-triggering experiments, bilayers were created using a 98:2 mixture of POPC:DGS-NTA-Ni^2+^ vesicles added to cleaned glass coverslips in 50-well silicon CultureWell covers (Grace Bio-Labs). Excess liposomes were removed by PBS washing and His-tagged proteins added afterward. Jurkat T cells were incubated with Fluo-4 dye (ThermoFisher) and then dropped onto prewarmed bilayers to measure Ca^2+^ flux by 488 fluorescence by confocal microscopy. For details of imaging and analysis code, refer to *SI Appendix*.

## Supplementary Material

Appendix 01 (PDF)

## Data Availability

All .fcs and .lsm5 files have been deposited in an OSF database (82). Analysis code is available for both the calcium analysis (https://github.com/janehumphrey/calcium) and the statistical analysis of sFCS data (https://github.com/Faldalf/sFCS_BTS). All other data are available in the manuscript and/or *SI Appendix*.
